# Supplementary material for: A chimeric viral platform for directed evolution in mammalian cells
Source: Nat Commun. 2025 May 7;16:4250. doi: 10.1038/s41467-025-59438-2 (PMC12059018; doi:10.1038/s41467-025-59438-2)
Supplement: Supplementary file 1 — Supplementary Information [file 41467_2025_59438_MOESM1_ESM.pdf]

A chimeric viral platform for directed evolution in mammalian cells

Supplementary Information

Alexander J. Cole[1†], Christopher E. Denes[2†], Cesar L. Moreno[2], Lise Hunault[1], Thomas Dobson[1], Daniel Hesselson[1*‡], G. Gregory Neely[2*‡]

Corresponding authors: d.hesselson@centenary.org.au (D.H); greg.neely@sydney.edu.au (G.G.N.);

The PDF file includes:

Supplementary Figures 1 to 9

Other Supplementary Information for this manuscript include the following:

Supplementary Data 1 to 7
Supplementary Movies 1 to 3

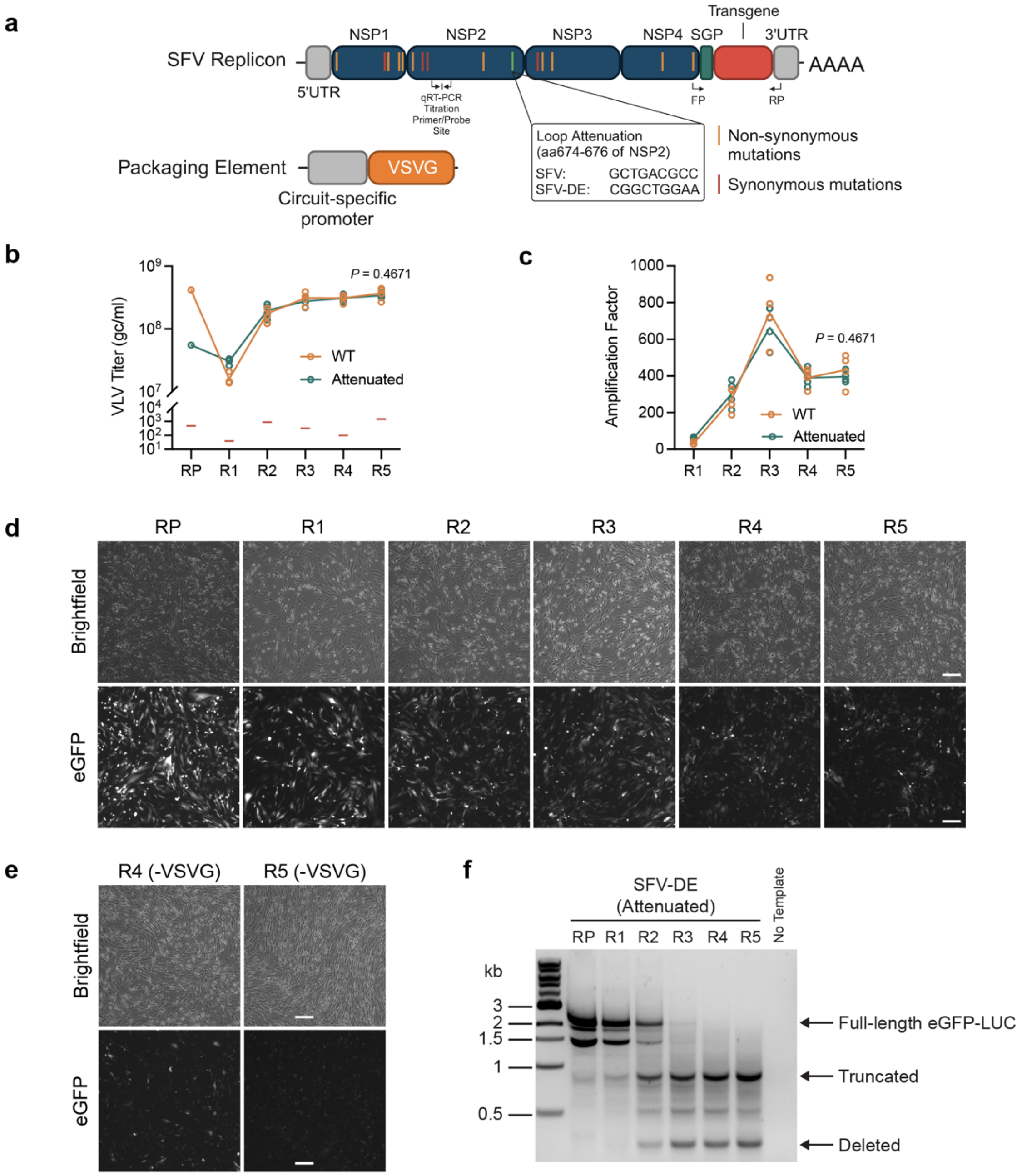

Supplementary Fig. 1. Benchmarking the PROTEUS platform. (a) Schematic representation of the PROTEUS SFV DNA replicon genome. FP/RP indicate the positions of the forward (FP) and reverse (RP) primers used for transgene isolation/sequencing. UTR = untranslated region; NSP = non-structural protein; SGP = sub-genomic promoter. Schematic was created with BioRender.com. Propagation **(b)** and amplification **(c)** of WT and ADA>RLE attenuated eGFP-LUC VLVs in CMV_VSVG-expressing BHK-21 cells for all rounds (R) RP-R5 ($N = 4$ biological replicates). Red bars indicate the RT-qPCR NTC signal in (**b**). Statistical comparisons were made using two-tailed unpaired t-tests. **(d)** Brightfield and epifluorescence microscopy of +VSVG BHK-21 cells at RP or following transduction with eGFP-LUC VLVs at RA-R4 (representative of $N = 4$ biological replicates). Scale bars, 200 µm. **(e)** Microscopy of -VSVG BHK-21 cells transduced at R3 and R4 (representative of $N = 4$ biological replicates). Scale bars, 200 µm. **(f)** RT-PCR and DNA gel electrophoresis of SFV-DE eGFP-LUC VLV transgenes ($N =$ 4 pooled biological replicates). Source data are provided as a Source Data file.

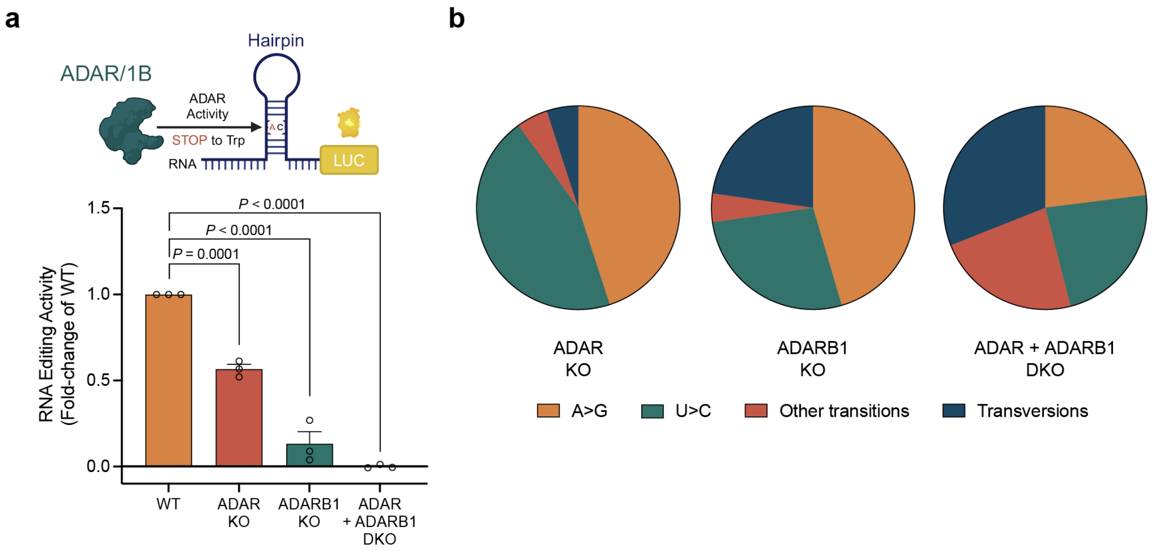

Supplementary Fig. 2. Mutational spectrum changes can be induced by ADAR knockout. (a) Reporter assay design (upper panel) and RNA editing activity of an ADAR biosensor in ADAR and/or ADARB1 knockout BHK-21 cells (lower panel) ($N = 3$ biological replicates). Error bars represent mean ± SEM. Statistical comparisons were made using an ordinary one-way ANOVA with Dunnett's multiple comparisons test to generate P values. Schematic was created with BioRender.com. **(b)** Mutational spectrum of eGFP-LUC variants derived from VLV propagation in ADAR and/or ADARB1 knockout BHK-21 cells. Source data are provided as a Source Data file.

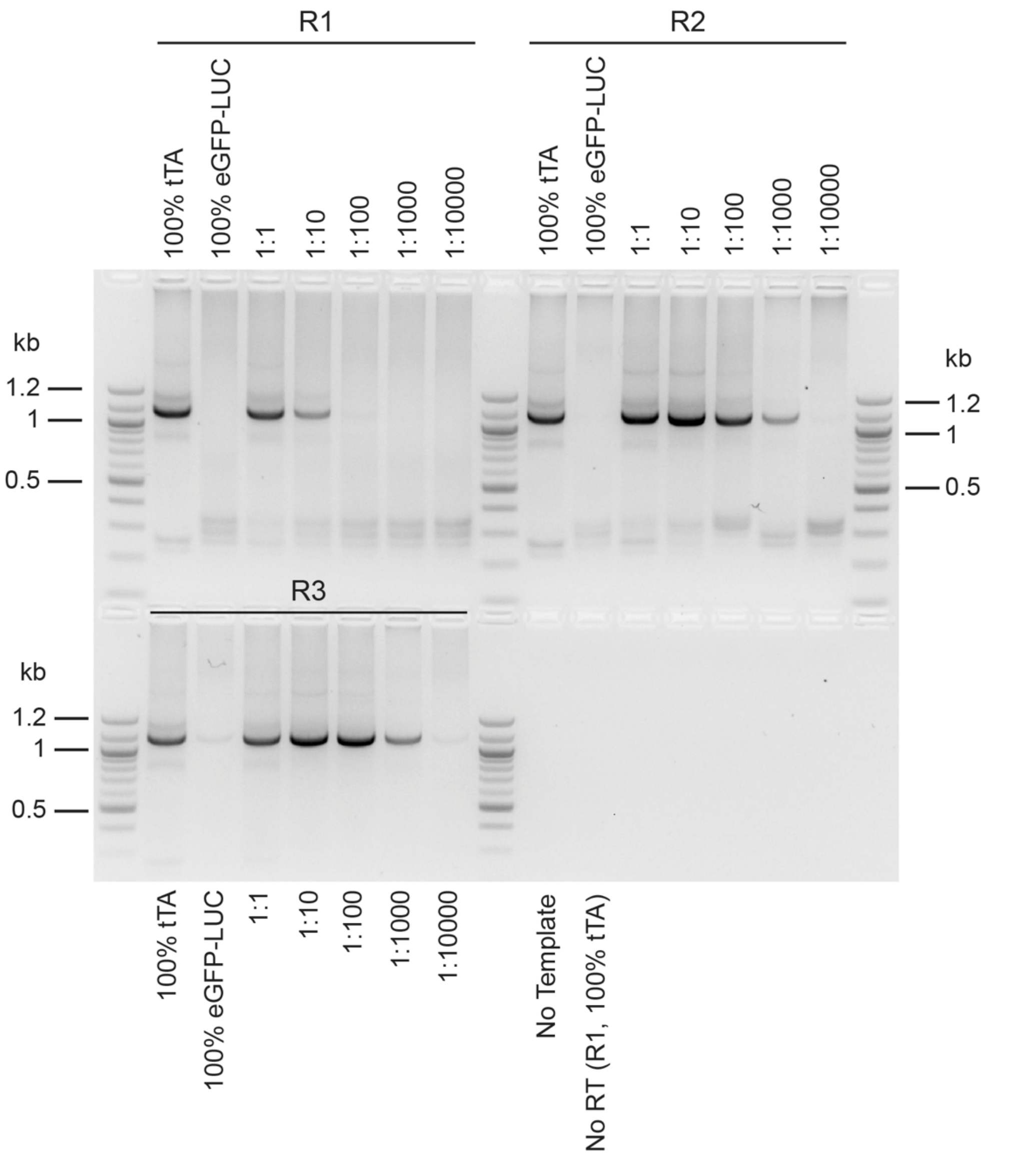

Supplementary Fig. 3. RT-PCR and DNA gel electrophoresis of isolated transgenes from serially diluted SFV-DE tTA : eGFP-LUC VLVs (R1-R3; *N* = 3 pooled). Source data are provided as a Source Data file.

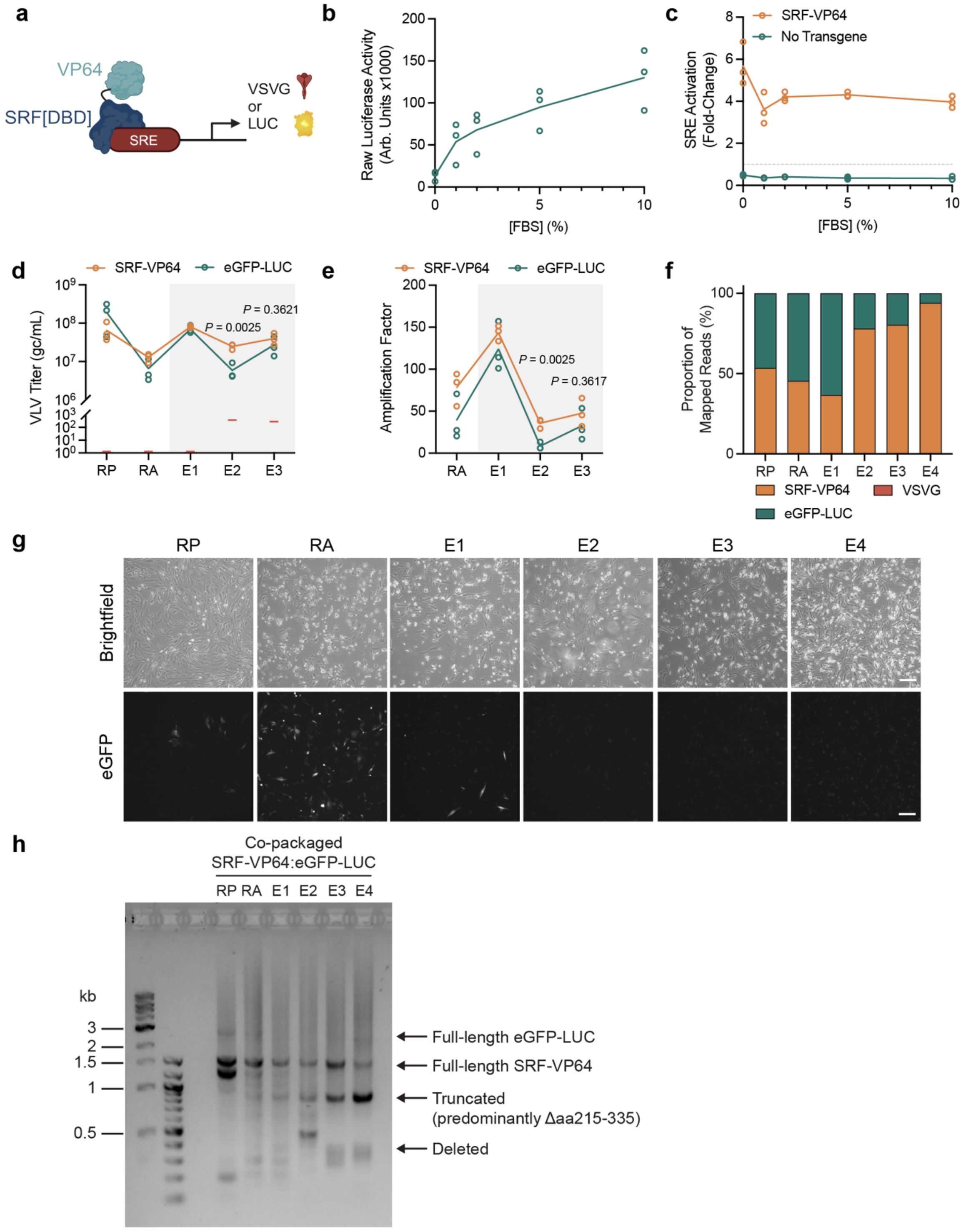
a
VP64
SRF[DBD]
SRE
VSVG
or
LUC
b
Raw Luciferase Activity
(Arb. Units x1000)
[FBS] (%)
c
SRF-VP64
No Transgene
SRE Activation
(Fold-Change)
[FBS] (%)
d
SRF-VP64
eGFP-LUC
VLV Titer (gc/mL)
P = 0.3621
P = 0.0025
RP
RA
E1
E2
E3
e
SRF-VP64
eGFP-LUC
Amplification Factor
P = 0.0025
P = 0.3617
RA
E1
E2
E3
f
Proportion of
Mapped Reads (%)
RP
RA
E1
E2
E3
E4
SRF-VP64
VSVG
eGFP-LUC
g
RP
RA
E1
E2
E3
E4
Brightfield
eGFP
h
Co-packaged
SRF-VP64:eGFP-LUC
RP RA E1 E2 E3 E4
kb
3
2
1.5
1
0.5
Full-length eGFP-LUC
Full-length SRF-VP64
Truncated
(predominantly Δaa215-335)
Deleted

i

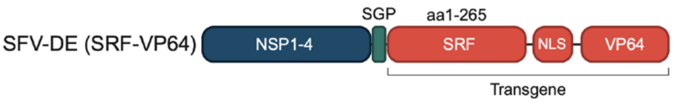

Protein Domain	Amino Acid Substitution	Nucleotide Substitution	Frequency (%) E3	Frequency (%) E4
SRF	RGS(aa14-16)> SGQRL and Δaa17-136	AGAGGCT>**TCTGGGC**AG AGGCTG (bold is inserted) AND deletion of coding sequence for Δaa17-136	81.82	90.91
	A6A (synonymous)	T>A	9.09	N.D.
	Q182R	A>G	N.D.	18.18
	A198A (synonymous)	C>T	N.D.	9.09
	R200R (synonymous)	G>A	N.D.	9.09
	C216R	T>C	9.09	N.D.
	N220D	A>G	N.D.	9.09
VP64	D5D (synonymous)	T>C	9.09	N.D.
	M10T	T>C	N.D.	9.09
	Sequence identified as derived from eGFP	N/A	9.09	9.09

j

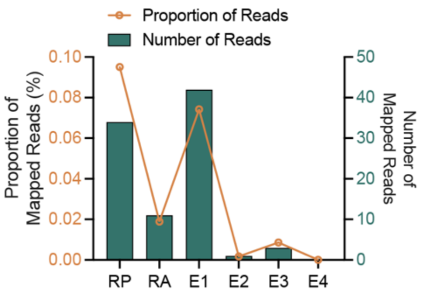

Supplementary Fig. 4. VLV propagation is dependent on host expression of VSVG. (a) Circuit design for SRF-VP64 mediated activation of SRE. **(b)** FBS dose-dependent SRE_LUC reporter activation by endogenous factors ($N = 3$ biological replicates). **(c)** SRF-VP64-mediated induction of an SRE-regulated LUC reporter ($N = 3$ biological replicates). Titers **(d)** and amplification factors **(e)** of SRF-VP64 and neutral eGFP-LUC VLVs propagated on cells expressing VSVG under the control of an SRE promoter in 1% FBS-supplemented growth medium (gray box) ($N = 3$ biological replicates). Red bars indicate the RT-qPCR NTC signal. Statistical comparisons were made using two-tailed unpaired t-tests. (**f**) Nanopore sequencing of transgene RNA isolated from pooled co-packaged VLV samples ($N = 6$ biological replicates) aligned to reference sequences (>10,000 reads per sample). For E1-E4, VLVs were propagated in 1% FBS-supplemented growth medium. **(g)** Brightfield and epifluorescence microscopy of BHK-21 cells at RP or following transduction with a 1:1 packaged cohort of SRF-VP64:eGFP-LUC VLVs at RA-E4 (representative of $N = 6$ biological replicates). Scale bars, 200 µm. **(h)** RT-PCR and DNA gel electrophoresis of isolated transgenes from a 1:1 packaged cohort of SRF-VP64:eGFP-LUC VLVs ($N = 6$ pooled biological replicates). (**i**) The DNA bands from E3 and E4 marked as 'Truncated' in **(h)** were cloned and sequenced ($N = 11$ clones per sample) to identify sequence domain and nucleotide substitutions. **(j)** Reads from **(f)** (>10,000 per sample) were aligned to a VSVG reference sequence. Schematics in panels **(a)** and **(i)** were created with BioRender.com. Source data are provided as a Source Data file.

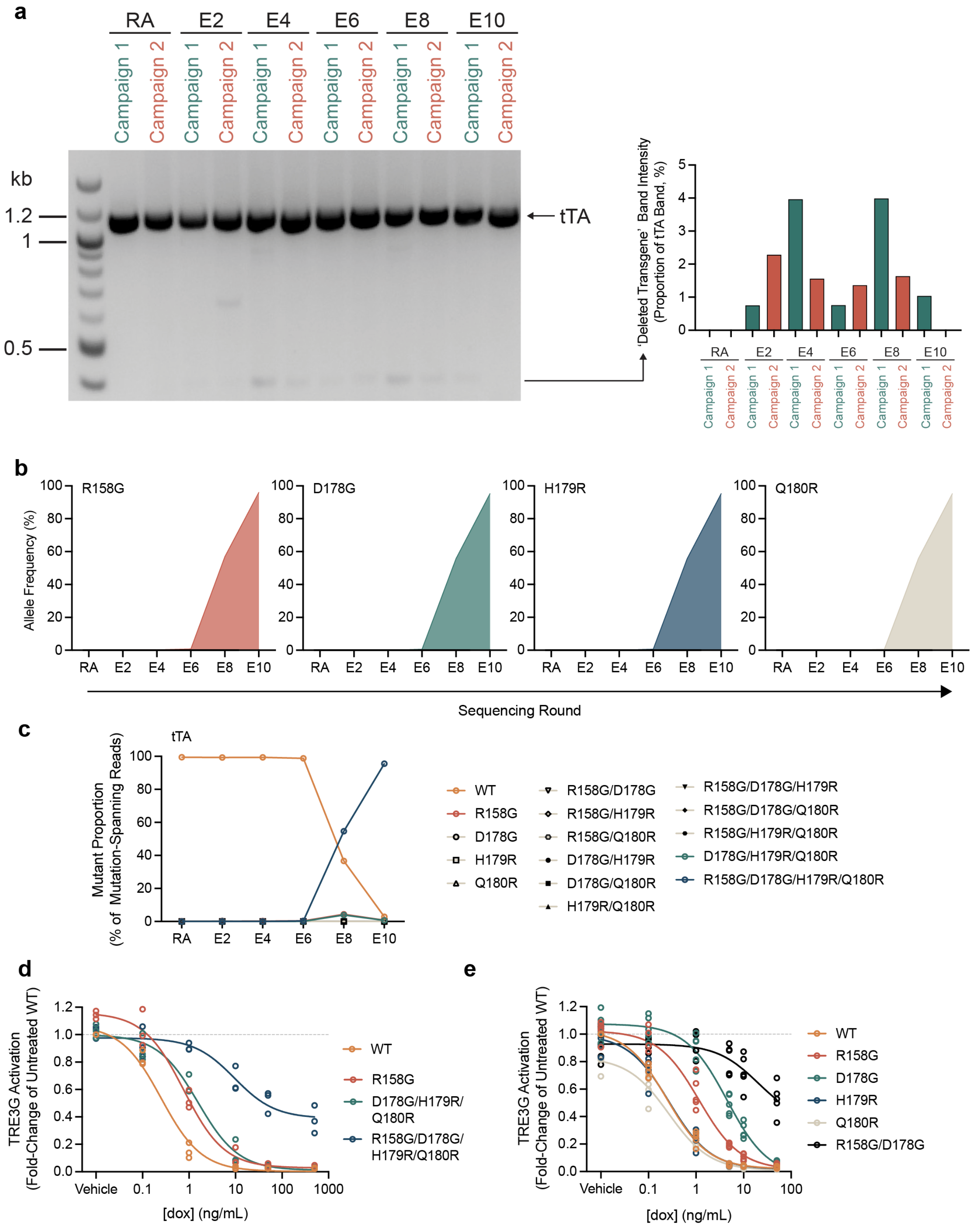
a
RA
E2
E4
E6
E8
E10
Campaign 1
Campaign 2
kb
1.2
1
0.5
tTA
'Deleted Transgene' Band Intensity (Proportion of tTA Band, %)
b
R158G
D178G
H179R
Q180R
Allele Frequency (%)
Sequencing Round
c
tTA
Mutant Proportion (% of Mutation-Spanning Reads)
WT
R158G
D178G
H179R
Q180R
R158G/D178G
R158G/H179R
R158G/Q180R
D178G/H179R
D178G/Q180R
H179R/Q180R
R158G/D178G/H179R
R158G/D178G/Q180R
R158G/H179R/Q180R
D178G/H179R/Q180R
R158G/D178G/H179R/Q180R
d
TRE3G Activation (Fold-Change of Untreated WT)
[dox] (ng/mL)
Vehicle
WT
R158G
D178G/H179R/Q180R
R158G/D178G/H179R/Q180R
e
TRE3G Activation (Fold-Change of Untreated WT)
[dox] (ng/mL)
WT
R158G
D178G
H179R
Q180R
R158G/D178G

f

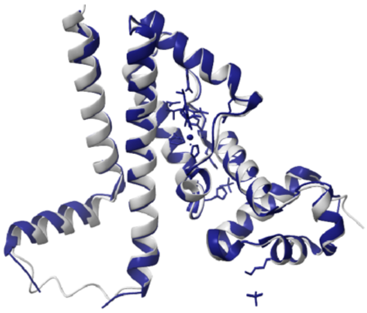

g

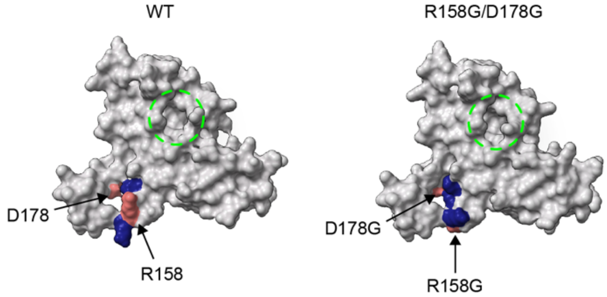

Supplementary Fig. 5. Directed evolution of tTA (Campaign 2). (a) DNA gel electrophoresis of isolated tTA transgenes following RT-PCR (from independent Campaigns 1 and 2). The proportion of amplicons that have lost the tTA transgene are calculated by densitometry. Allele frequency of the major variants individually **(b)** and combined **(c)** identified in Campaign 2. A similar analysis of mutation combinations for Campaign 1 cannot be extracted from short-read Illumina sequencing because of the distance between the identified Q32R and R158G residues. Note, a synonymous passenger mutation (E159E) rose to similar levels as each of the R158G, D178G, H179R, Q180R mutations during this campaign. Analysis for **(c)** accounted for synonymous substitutions at E159 to capture all reads analyzed in **(b)**. **(d)** Dox-resistance of evolved tTA variants (*N* = 5 biological replicates). **(e)** Isolated effects of aa178-180 single mutants (*N* = 5 biological replicates). **(f)** Alignment of crystal structure 4AC0 (blue) with Alphafold2-modeled tTA (gray). **(g)** Variant-induced structural changes in tTA modeled with AlphaFold2 (red, mutated residues; blue, displaced functional groups; dashed green circle, drug binding pocket). Source data are provided as a Source Data file.

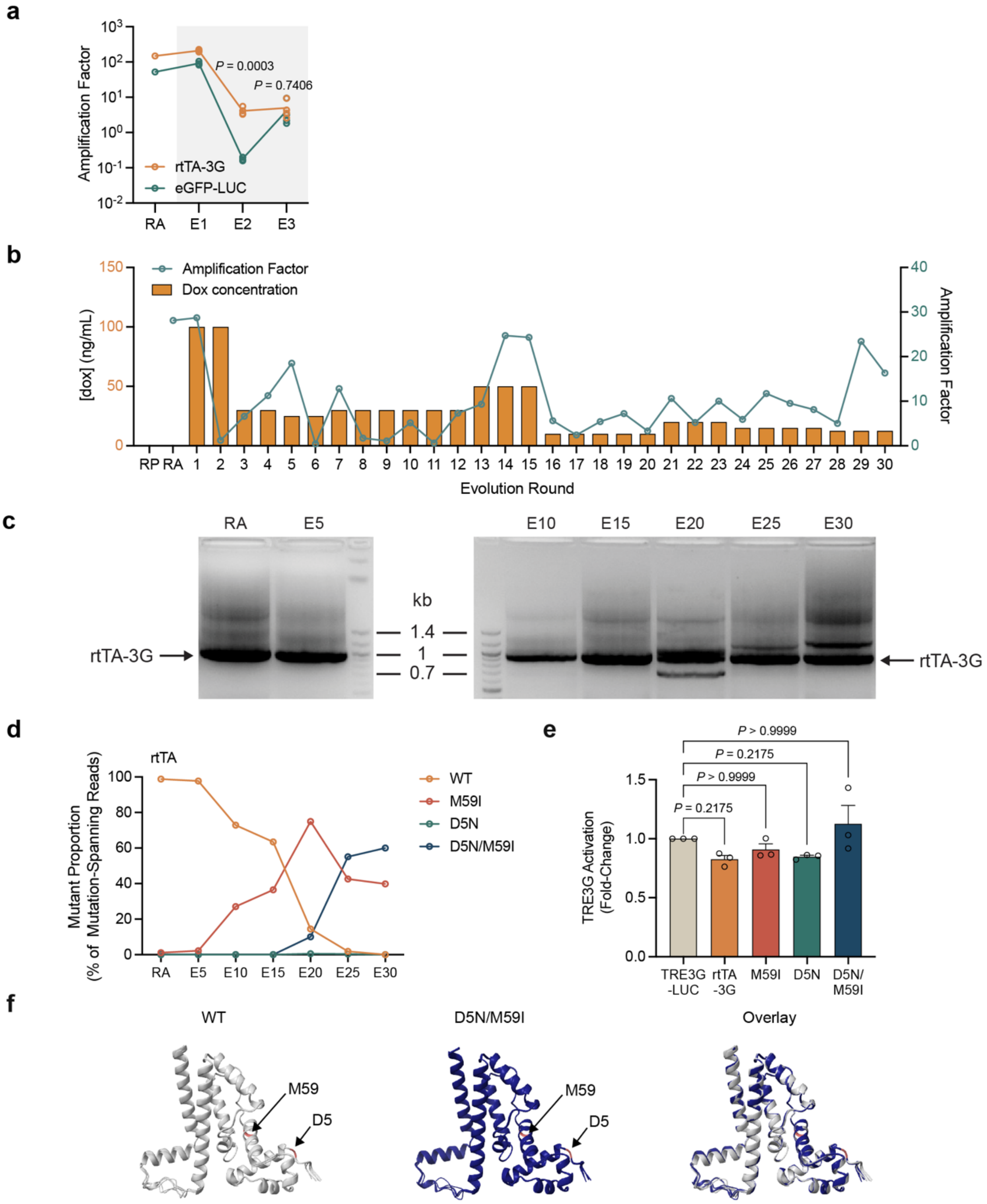

Supplementary Fig. 6. Directed evolution of rtTA-3G. (a) Amplification factors of VLVs propagated on cells expressing VSVG under the control of TRE3G at 100 ng/mL dox (gray box) ($N = 4$ biological replicates). Statistical comparisons were made using two-tailed unpaired t-tests. **(b)** Dox concentration and AF for each round of rtTA-3G evolution. **(c)** DNA gel electrophoresis of isolated rtTA transgenes following RT-PCR. **(d)** Allele frequencies of single and double mutant variants during long-term propagation on minimal concentrations of dox. **(e)** Basal activity of the evolved variants in the absence of dox ($N = 3$ biological replicates). Error bars represent mean ± SEM. Statistical comparisons were made using a Kruskal-Wallis test with Dunn's multiple comparisons test to generate P values. **(f)** Alignment of top 5 ranked Alphafold2 predictions for rtTA-3G (gray) and the D5N/M59I variant (blue; red, mutated residues). Source data are provided as a Source Data file.

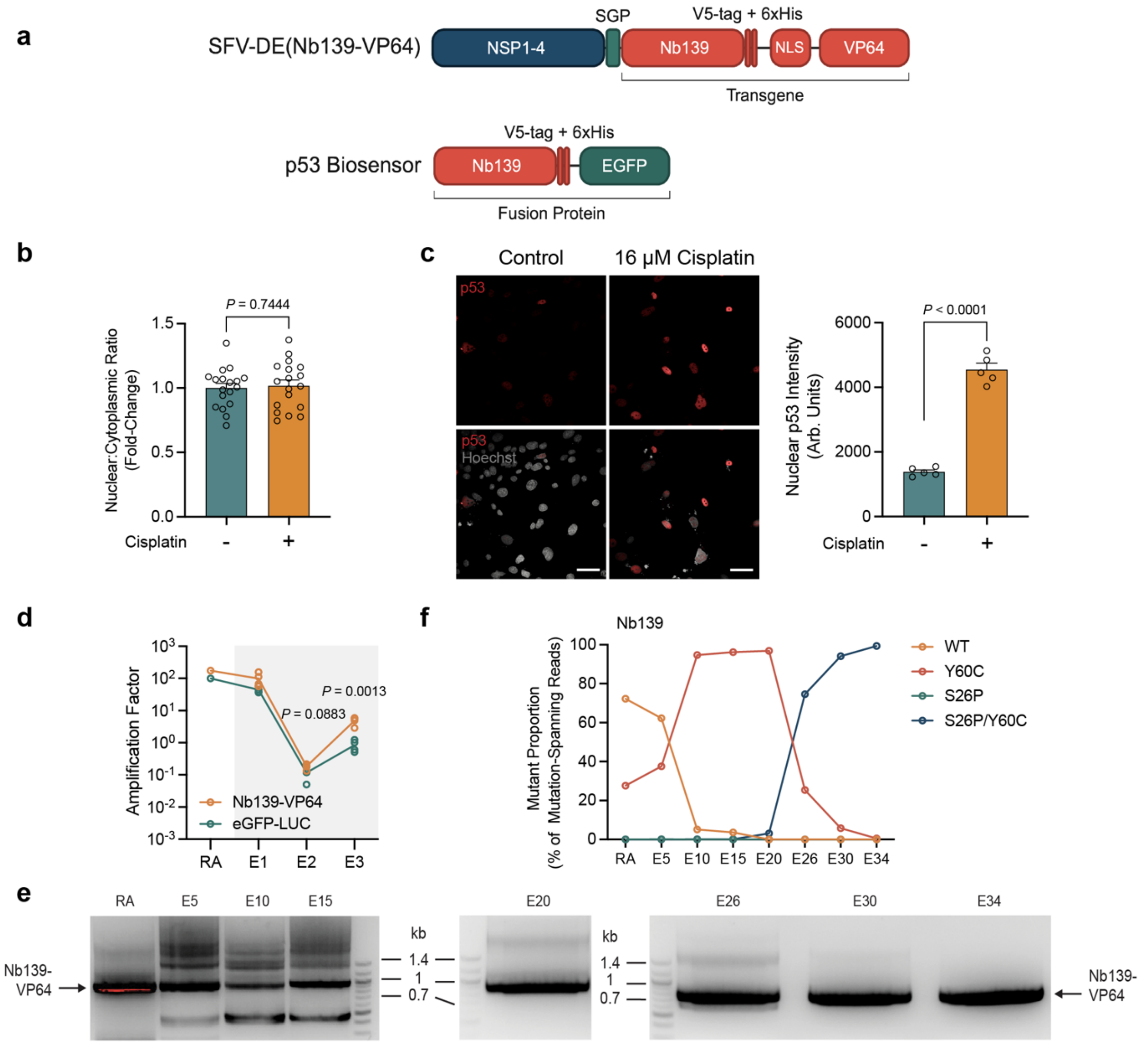

Supplementary Fig. 7. Directed evolution of a nanobody with PROTEUS. (a) Schematic of the Nb139-VP64 SFV-DE evolutionary construct and Nb139-eGFP biosensor design. The p53 biosensor construct does not contain an NLS. Schematic was created with BioRender.com. **(b)** Effect of 16 µM cisplatin on parental Nb139-eGFP nuclear localization in BHK-21 cells ($N = 18$ measured cells). Error bars represent mean ± SEM. Statistical comparisons were made using an unpaired two-tailed t-test. **(c)** p53 immunofluorescence (red) and Hoechst nuclear staining (gray) and quantification of nuclear p53 accumulation in BHK-21 cells. Scale bars, 50 µm. ($N \geq 272$ cells analyzed per replicate; images are representative of $N = 5$ biological replicates). Error bars represent mean ± SEM. Statistical comparisons were made using a paired two-tailed t-test. **(d)** Amplification factors of VLVs propagated on cells expressing VSVG under the control of a p53 2-hybrid circuit (gray box) ($N = 4$ biological replicates). Statistical comparisons were made using an unpaired two-tailed t-test. **(e)** DNA gel electrophoresis of isolated Nb139-VP64 transgenes following RT-PCR. **(f)** Allele frequencies of single and double mutant variants during long-term propagation on the 2-hybrid circuit. Source data are provided as a Source Data file.

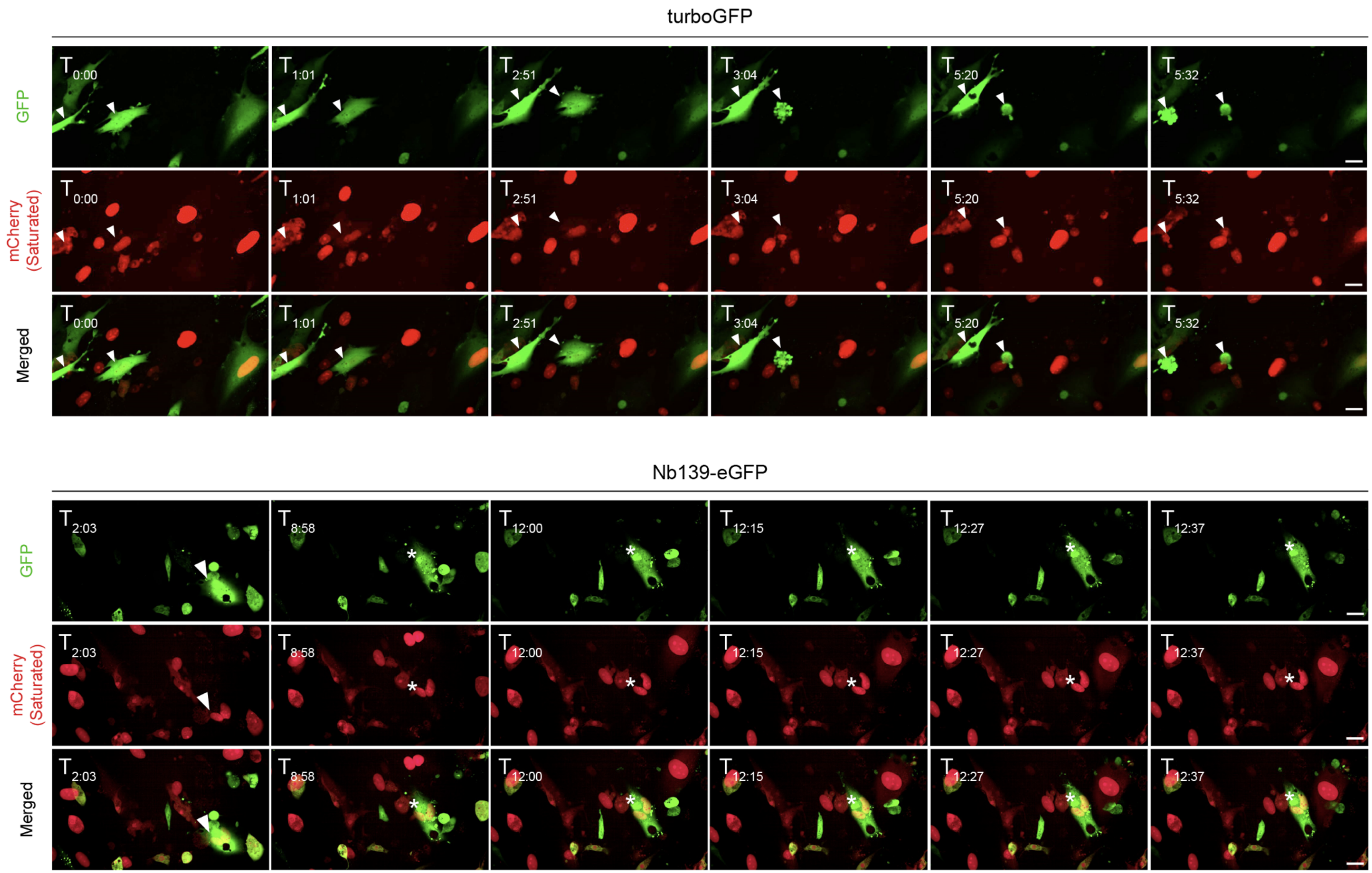
turboGFP
GFP
mCherry (Saturated)
Merged
T 0:00
T 1:01
T 2:51
T 3:04
T 5:20
T 5:32
Nb139-eGFP
T 2:03
T 8:58
T 12:00
T 12:15
T 12:27
T 12:37

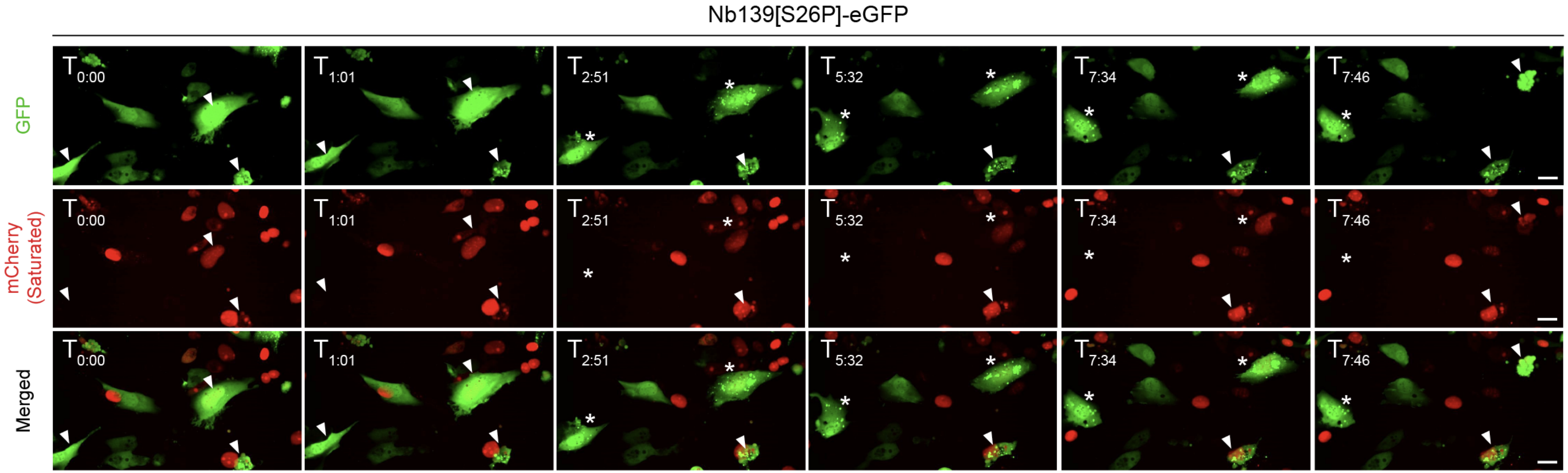

Supplementary Fig. 8. Timelapse of cisplatin-treated cells that express parental Nb139-eGFP, Nb139[S26P]-eGFP fusion or turboGFP alone. Individual channels from timelapse in **Fig. 4g**. Nuclei labeled with mCherry (red). TurboGFP control or Nb139-eGFP and Nb139[S26P]-eGFP biosensors (green). White arrows indicate cells of interest; asterisks indicate foci formation. Scale bars, 25 µm. The p53 biosensor construct does not contain an NLS. Representative of $N = 2$ biological replicates.

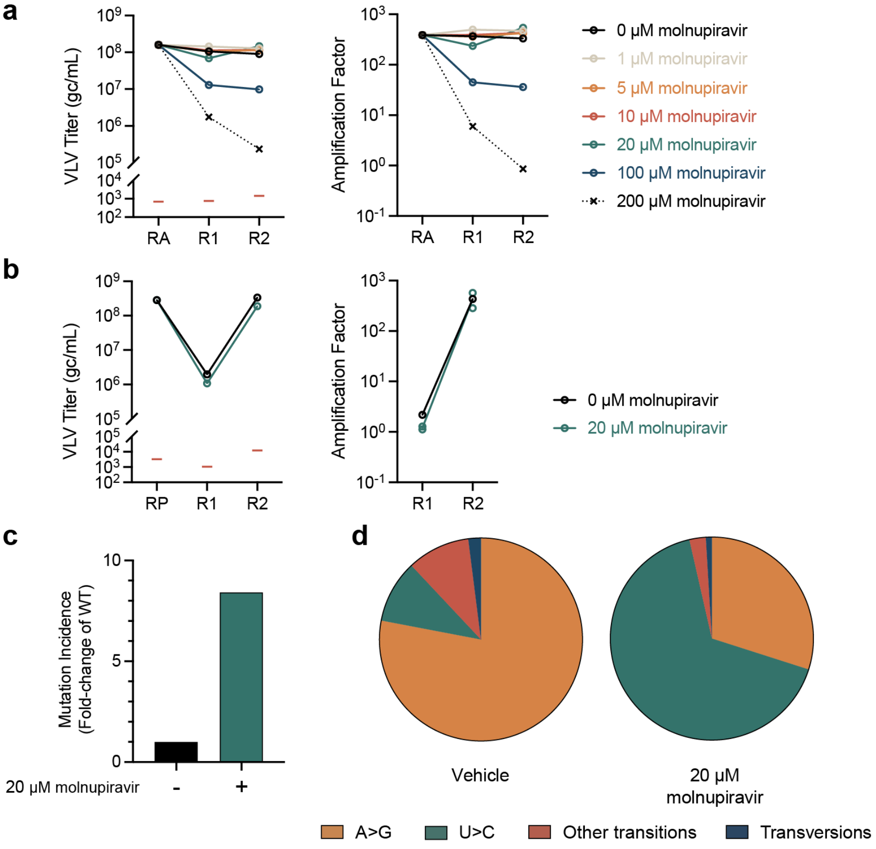

Supplementary Fig. 9. Mutational spectrum changes can be induced by ADAR knockout and nucleoside analog supplementation. **(a)** Titers and amplification factors of eGFP-LUC VLVs propagated in BHK-21 cells constitutively expressing CMV_VSVG for rounds RA-R2 in the presence of escalating molnupiravir concentrations (*N* = 1 biological replicate). Red bars indicate the RT-qPCR NTC signal. **(b)** Titers and amplification factors of eGFP-LUC VLVs propagated in cells constitutively expressing CMV_VSVG for rounds RP-R2 ± 20 μM molnupiravir (*N* = 2 biological replicates). **(c)** Mutations detected in VLVs propagated ± molnupiravir (*N* = 2 biological replicates from **(b)**, pooled prior to viral RNA extraction, transgene isolation and next generation sequencing). **(d)** Mutational spectrum of viral variants propagated on BHK-21 cells ± 20 μM molnupiravir. Source data are provided as a Source Data file.
