# Supplementary material for: A chimeric viral platform for directed evolution in mammalian cells
Source: Nat Commun. 2025 May 7;16:4250. doi: 10.1038/s41467-025-59438-2 (PMC12059018; doi:10.1038/s41467-025-59438-2)
Supplement: Supplementary file 2 — Reporting Summary [file 41467_2025_59438_MOESM2_ESM.pdf]

nature portfolio

Corresponding author(s): Neely GG

Last updated by author(s): Denes CE

Reporting Summary

Nature Portfolio wishes to improve the reproducibility of the work that we publish. This form provides structure for consistency and transparency in reporting. For further information on Nature Portfolio policies, see our Editorial Policies and the Editorial Policy Checklist.

Statistics

For all statistical analyses, confirm that the following items are present in the figure legend, table legend, main text, or Methods section.

n/a	Confirmed	
☐	☒	The exact sample size (*n*) for each experimental group/condition, given as a discrete number and unit of measurement
☐	☒	A statement on whether measurements were taken from distinct samples or whether the same sample was measured repeatedly
☐	☒	The statistical test(s) used AND whether they are one- or two-sided<br>*Only common tests should be described solely by name; describe more complex techniques in the Methods section.*
☒	☐	A description of all covariates tested
☐	☒	A description of any assumptions or corrections, such as tests of normality and adjustment for multiple comparisons
☐	☒	A full description of the statistical parameters including central tendency (e.g. means) or other basic estimates (e.g. regression coefficient) AND variation (e.g. standard deviation) or associated estimates of uncertainty (e.g. confidence intervals)
☐	☒	For null hypothesis testing, the test statistic (e.g. *F*, *t*, *r*) with confidence intervals, effect sizes, degrees of freedom and *P* value noted<br>*Give P values as exact values whenever suitable.*
☒	☐	For Bayesian analysis, information on the choice of priors and Markov chain Monte Carlo settings
☒	☐	For hierarchical and complex designs, identification of the appropriate level for tests and full reporting of outcomes
☒	☐	Estimates of effect sizes (e.g. Cohen's *d*, Pearson's *r*), indicating how they were calculated

Our web collection on statistics for biologists contains articles on many of the points above.

Software and code

Policy information about availability of computer code

Data collection	Brightfield and EGFP epifluorescence images were obtained on an Axio Vert.A1 FL (Zeiss) fitted with an AxioCam ICM1 camera (Zeiss 60N-C 2/3" 0.63X adapter) at 5X magnification with the software Zen 2 Blue Edition (Zeiss, version 2.0.0.0). High-throughput imaging data was captured using the Opera Phenix Plus High-Content Screening system. Short-read sequencing data was collected on an Illumina NovaSeq 6000 using NovaSeq PE250 technology. Long-read nanopore sequencing data was collected on an Oxford Nanopore Flongle flow cell (R9.4.1 chemistry, #FLO-FLG001) in a MinION Mk1B Sequencer (ONT, #MIN-101B) fitted with a Flongle Adapter (ONT, #ADP-FLG001). RT-qPCR titration data was collected in 384-well plates using a QuantStudio™ 7 Flex or QuantStudio™ 6 Pro Real-Time PCR System (ThermoFisher). mCherry expression in bacterial cells was measured using a BD LSR II cell analyzer (BD Biosciences).
Data analysis	Sanger sequencing alignment analysis was performed in SnapGene (Version 6.1 or greater). RT-qPCR data was analysed using QuantStudio™ Real-Time PCR Software v1.3 (QuantStudio 7 Flex) or Design and Analysis 2.6.0 (QuantStudio 6 Pro) from ThermoFisher. High-throughput imaging data was analysed with Harmony Software V5.1 (Perkin Elmer). Long-read nanopore sequencing was aligned to reference sequences using EPI2ME Desktop Agent (ONT, version 3.3.0.1031). Short-read sequencing was analysed for variant nucleotides with minimap2, samtools, VarScan 2.3.9 and vcf-annotator. Mutation co-occurrence was analysed using the simple 'grep' command. Statistical analyses were performed in GraphPad Prism 9.2.0 or greater. Flow cytometry data was analysed with FlowJo (v10.10).

For manuscripts utilizing custom algorithms or software that are central to the research but not yet described in published literature, software must be made available to editors and reviewers. We strongly encourage code deposition in a community repository (e.g. GitHub). See the Nature Portfolio guidelines for submitting code & software for further information.

Data

Policy information about availability of data

All manuscripts must include a data availability statement. This statement should provide the following information, where applicable:

- Accession codes, unique identifiers, or web links for publicly available datasets
- A description of any restrictions on data availability
- For clinical datasets or third party data, please ensure that the statement adheres to our policy

The authors confirm that the data supporting findings of this study are available within the article and its supplementary materials. Basecalled long-read nanopore sequencing FASTQ reads and raw short-read Illumina sequencing FASTQ reads have been deposited at the Gene Expression Omnibus (GEO; GSE250502). PROTEUS plasmids will be made available on Addgene for academic use.

Research involving human participants, their data, or biological material

Policy information about studies with human participants or human data. See also policy information about sex, gender (identity/presentation), and sexual orientation and race, ethnicity and racism.

Reporting on sex and gender	N/A
Reporting on race, ethnicity, or other socially relevant groupings	N/A
Population characteristics	N/A
Recruitment	N/A
Ethics oversight	N/A

Note that full information on the approval of the study protocol must also be provided in the manuscript.

Field-specific reporting

Please select the one below that is the best fit for your research. If you are not sure, read the appropriate sections before making your selection.

☒ Life sciences ☐ Behavioural & social sciences ☐ Ecological, evolutionary & environmental sciences

For a reference copy of the document with all sections, see nature.com/documents/nr-reporting-summary-flat.pdf

Life sciences study design

All studies must disclose on these points even when the disclosure is negative.

Sample size	Statistical methods were not used to predetermine sample size. For each assay reported, typically three biological replicates were performed, except for evolution studies where 1 to 2 independent evolution campaigns were performed, with subsequent validation and replication of the effects of dominating variant sequences.
Data exclusions	No data were excluded.
Replication	Individual data points are represented where appropriate in all figures, with sample size indicated in the figure legends.
Randomization	Randomization is not relevant to this study as experiments were performed using cell lines or bacterial strains.
Blinding	Blinding was not relevant to this study as experiments did not require subjective analysis.

Reporting for specific materials, systems and methods

We require information from authors about some types of materials, experimental systems and methods used in many studies. Here, indicate whether each material, system or method listed is relevant to your study. If you are not sure if a list item applies to your research, read the appropriate section before selecting a response.

Materials & experimental systems

n/a	Involved in the study
☐	☒ Antibodies
☐	☒ Eukaryotic cell lines
☒	☐ Palaeontology and archaeology
☒	☐ Animals and other organisms
☒	☐ Clinical data
☒	☐ Dual use research of concern
☒	☐ Plants

Methods

n/a	Involved in the study
☒	☐ ChIP-seq
☐	☒ Flow cytometry
☒	☐ MRI-based neuroimaging

Antibodies

Antibodies used	p53 Rabbit mAb (Clone 7F5), Cell Signaling Technology (Catalog #CST2527), lot 12<br>Goat anti-Rabbit IgG (H+L) Highly Cross-Adsorbed Secondary Antibody, Alexa Fluor™ 647, Invitrogen (Catalog #A21245), lot 1922848
Validation	p53 Rabbit mAb (Clone 7F5): The manufacturer's website demonstrates specificity of the anti-p53 primary antibody for westerm blot, immunohistochemistry, immunofluorescence, flow cytometry and ChIP. Western blotting images of cells containing p53 show a strong single band at 53 kDa with no additional nonspecific bands. No staining is observed in p53-deficient cells.<br>Goat anti-Rabbit IgG (H+L) Highly Cross-Adsorbed Secondary Antibody, Alexa Fluor™ 647: from the website: 'To minimize cross-reactivity, these goat anti-rabbit IgG whole antibodies have been cross-adsorbed against bovine IgG, goat IgG, mouse IgG, rat IgG, and human IgG.'

Eukaryotic cell lines

Policy information about cell lines and Sex and Gender in Research

Cell line source(s)	BHK-21 [C-13] cells were sourced from the American Type Culture Collection (#CCL-10). These animal cells were derived from baby hamster kidneys of five unsexed, 1-day-old hamsters (Mesocricetus auratus) in March, 1961, by I.A. Macpherson and M.G.P. Stoker. Induced pluripotent stem cells were sourced from Stem Cell Technologies (#SCTi003-A). These cells were derived from a healthy human female donor's peripheral blood mononuclear cells. HEK293T cells were sourced from the American Type Culture Collection (#CRL-3216). These epithelial human (Homo sapiens) cells were derived from a female embryonic kidney.
Authentication	Cell lines were not further authenticated.
Mycoplasma contamination	Cells tested negative for mycoplasma.
Commonly misidentified lines (See ICLAC register)	No commonly misidentified cell lines were used in this study.

Plants

Seed stocks	N/A
Novel plant genotypes	N/A
Authentication	N/A

Flow Cytometry

Plots

Confirm that:

☒ The axis labels state the marker and fluorochrome used (e.g. CD4-FITC).

☒ The axis scales are clearly visible. Include numbers along axes only for bottom left plot of group (a 'group' is an analysis of identical markers).

☒ All plots are contour plots with outliers or pseudocolor plots.

☒ A numerical value for number of cells or percentage (with statistics) is provided.

Methodology

Sample preparation	Following experiments, E. coli bacteria were fixed in Fixation Buffer (Flow Cytometry) (Abcam, #ab314680) under manufacturer's conditions.
Instrument	BD LSR II cell analyzer (BD Biosciences)
Software	BD FACS Diva 8.0.2
Cell population abundance	In all events, the singlets were selected, accounting for 97.6% of the population. In those, the bacteria population was selected based on FSC and accounted for 91.8% of the population. mCherry fluorescence was read in that sub-population.
Gating strategy	In all events, the singlets were selected, followed by the bacteria population based on FSC. In that population mCherry fluorescence was assessed. This information is presented in the Source Data for Figure 3g.

☒ Tick this box to confirm that a figure exemplifying the gating strategy is provided in the Supplementary Information.
