# Supplementary material for: A chimeric viral platform for directed evolution in mammalian cells
Source: Nat Commun. 2025 May 7;16:4250. doi: 10.1038/s41467-025-59438-2 (PMC12059018; doi:10.1038/s41467-025-59438-2)
Supplement: Supplementary file 13 — Description of Additional Supplementary Files [file 41467_2025_59438_MOESM13_ESM.pdf]

Legends for Supplementary Data and Supplementary Movies

Supplementary Data 1. eGFP-LUC variants. All substitutions in transgene RNA detected at ≥0.3% over three rounds of VLV propagation.

Supplementary Data 2. tTA variants. Non-synonymous substitutions in transgene RNA detected at ≥1% during two PROTEUS campaigns.

Supplementary Data 3. Analysis of tTA and rtTA-3G mutants. Half maximal inhibitory/effective concentrations (IC50/EC50) and 95% confidence intervals (95% CI) for tTA and rtTA-3G figures.

Supplementary Data 4. rtTA-3G variants. Non-synonymous substitutions in transgene RNA detected at ≥1% of the population during a PROTEUS campaign.

Supplementary Data 5. Nb139 variants. Non-synonymous substitutions in transgene RNA detected at ≥1% during a PROTEUS campaign.

Supplementary Data 6. List of plasmids used or generated in this study.

Supplementary Data 7. List of PROTEUS oligos.

Supplementary Movie 1. Videos for stills of turboGFP timelapse in Fig. 4G and Extended Data Fig. 8. turboGFP control in green. Nuclei labeled with mCherry (red).

Supplementary Movie 2. Videos for stills of Nb139-eGFP timelapse in Fig. 4G and Extended Data Fig. 8. Nb139-eGFP biosensor in green. Nuclei labeled with mCherry (red).

Supplementary Movie 3. Videos for stills of Nb139[S26P]-eGFP timelapse in Fig. 4G and Extended Data Fig. 8. Nb139[S26P]-eGFP biosensor in green. Nuclei labeled with mCherry (red).
